# Supplementary material for: Prevalence of Gestational Diabetes in Triplet Pregnancies: A Retrospective Cohort Study and Meta-Analysis
Source: J Clin Med. 2020 May 18;9(5):1523. doi: 10.3390/jcm9051523 (PMC7290297; doi:10.3390/jcm9051523)
Supplement: Supplementary file 1 [file jcm-09-01523-s001.pdf]

Table S1. Estimates and 95% confidence intervals obtained in a leave-one-out sensitivity analysis of all 38 eligible studies. The calculations are performed as in the main analysis. However, in each round of the sensitivity analysis one study is not included.

	Estimate Pooled	95% Confidence Interval
All studies included	0.073	0.050;0.099
Without Adesiyun & Eseigbe[25]	0.075	0.051;0.101
Without Almeida et al.[26]	0.072	0.049;0.098
Without Combs et al.[27]	0.071	0.048;0.097
Without Eddib et al.[28]	0.074	0.051;0.101
Without Elliott et al.[29]	0.076	0.053;0.102
Without Fennessy et al.[30]	0.073	0.049;0.099
Without Fitzsimmons et al.[31]	0.071	0.048;0.097
Without Ghazeeri et al.[23]	0.073	0.045;0.099
Without Guilherme et al.[32]	0.073	0.049;0.099
Without Hager et al.	0.067	0.047;0.091
Without Kawaguchi et al.[33]	0.077	0.054;0.103
Without Lipitz et al.[34]	0.074	0.050;0.100
Without Luke and Brown[22]	0.075	0.051;0.101
Without Machtinger et al.[35]	0.073	0.050;0.100
Without Macones et al.[36]	0.075	0.051;0.101
Without Makhseed et al.[37]	0.072	0.049;0.098
Without Malone et al.[38]	0.073	0.050;0.100
Without Manzur et al.[39]	0.072	0.049;0.099
Without Marino et al.[24]	0.074	0.050;0.100
Without Okyay et al.[40]	0.072	0.049;0.098
Without Pakrashi and Defranco[41]	0.070	0.048;0.096
Without Parkinson et al.[42]	0.073	0.050;0.099
Without Peress et a.[43]	0.074	0.050;0.101
Without Razaz et al.[44]	0.070	0.048;0.096
Without Revello et al.[45]	0.070	0.048;0.096
Without Salihu et al.[46]	0.075	0.051;0.102
Without Santema et al.[47]	0.074	0.051;0.101
Without Sato et al.[48]	0.076	0.054;0.102
Without Seoud et al.[49]	0.070	0.048;0.094
Without Simoes et al.[50]	0.075	0.051;0.101
Without Sivan et al.[9]	0.069	0.047;0.094
Without Sklar et al.[51]	0.071	0.048;0.097
Without Skrablin et al.[52]	0.076	0.053;0.102
Without Smith et al.[53]	0.073	0.050;0.100
Without Smith-Levitinet et al.[54]	0.072	0.049;0.098
Without Sumners et al.[55]	0.073	0.049;0.100
Without Weissman and Drugan[56]	0.072	0.049;0.098
Without Ziadeh et al.[57]	0.0749	0.052;0.101

Table S2. Estimates and 95% confidence intervals obtained in a leave-one-out sensitivity analysis of all 11 studies with a sound GDM definition. The calculations are performed as in the main analysis. However, in each round of the sensitivity analysis one study is not included.

	Estimate Pooled	95% Confidence Interval
All studies included	0.073	0.050;0.099
Without Fennessy et al.[30]	0.128	0.067;0.203
Without Hager et al.	0.107	0.060;0.165
Without Lipitz et al.[34]	0.131	0.070;0.206
Without Malone et al.[38]	0.131	0.070;0.205
Without Okyay et al.[40]	0.124	0.064;0.198

Without Revello et al.[45]	0.120	0.061;0.194
Without Seoud et al.[49]	0.112	0.061;0.174
Without Simoes et al.[50]	0.136	0.076;0.208
Without Sivan et al.[9]	0.115	0.060;0.184
Without Skrablin et al.[52]	0.141	0.088;0.204
Without Weissman and Drugan[56]	0.124	0.065;0.198

GDM, gestational diabetes mellitus.
